# Crystal structure of 1-(cyclo­pentyl­idene­amino)-3-(prop-2-en-1-yl)thio­urea

**DOI:** 10.1107/S2056989015021003

**Published:** 2015-11-11

**Authors:** Shaaban K. Mohamed, Joel T. Mague, Mehmet Akkurt, Alaa A. Hassan, Ahmed T. Abdel-Aziz, Mustafa R. Albayati

**Affiliations:** a Faculty of Science & Engineering, School of Healthcare Science, Manchester Metropolitan University, Manchester M1 5GD, England; b Chemistry Department, Faculty of Science, Minia University, 61519 El-Minia, Egypt; c Department of Chemistry, Tulane University, New Orleans, LA 70118, USA; d Department of Physics, Faculty of Sciences, Erciyes University, 38039 Kayseri, Turkey; e Kirkuk University, College of Education, Department of Chemistry, Kirkuk, Iraq

**Keywords:** crystal structure, thio­semicarbazides, hydrogen bonding

## Abstract

In the title compound, C_9_H_15_N_3_S, the cyclo­pentyl ring adopts an envelope conformation with one of the methyl­ene C atoms as the flap. The thio­semicarbazide fragment is almost planar (r.m.s. deviation = 0.038 Å) and a short intra­molecular N—H⋯N contact occurs. In the crystal, mol­ecules are linked into helical (4_1_ symmetry) chains propagating in [001] by N—H⋯N and N—H⋯S hydrogen bonds. A very weak C—H⋯S inter­action is also observed.

## Related literature   

For the biological activities of thio­semicarbazide-containing compounds, see: Hu *et al.* (2010 ▸); da Costa *et al.* (2015 ▸). For the synthesis of the title compound, see: Mague *et al.* (2014 ▸).
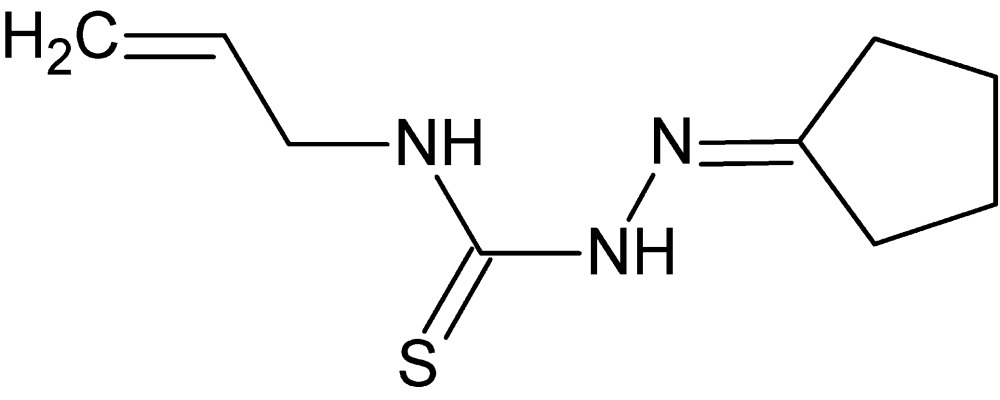


## Experimental   

### Crystal data  

C_9_H_15_N_3_S
*M* *_r_* = 197.30
Tetragonal, 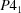

*a* = 9.0124 (2) Å
*c* = 12.8200 (2) Å
*V* = 1041.28 (5) Å^3^
*Z* = 4
Cu *K*α radiation
μ = 2.42 mm^−1^
*T* = 150 K
0.21 × 0.16 × 0.10 mm

### Data collection  

Bruker D8 VENTURE PHOTON 100 CMOS diffractometer
Absorption correction: multi-scan (*SADABS*; Bruker, 2014 ▸) *T*_min_ = 0.67, *T*_max_ = 0.79
7869 measured reflections
1990 independent reflections
1901 reflections with *I* > 2σ(*I*)
*R*_int_ = 0.032

### Refinement  

*R*[*F* ^2^ > 2σ(*F* ^2^)] = 0.041
*wR*(*F* ^2^) = 0.109
*S* = 1.10
1990 reflections
119 parameters
1 restraint
H-atom parameters constrained
Δρ_max_ = 0.70 e Å^−3^
Δρ_min_ = −0.18 e Å^−3^
Absolute structure: Flack *x* determined using 826 quotients [(*I* ^+^)−(*I* ^−^)]/[(*I* ^+^)+(*I* ^−^)] (Parsons *et al.*, 2013 ▸)
Absolute structure parameter: 0.04 (3)

### 

Data collection: *APEX2* (Bruker, 2014 ▸); cell refinement: *SAINT* (Bruker, 2014 ▸); data reduction: *SAINT*; program(s) used to solve structure: *SHELXT* (Sheldrick, 2015*a* ▸); program(s) used to refine structure: *SHELXL2014* (Sheldrick, 2015*b* ▸); molecular graphics: *DIAMOND* (Brandenburg & Putz, 2012 ▸); software used to prepare material for publication: *SHELXTL* (Sheldrick, 2008 ▸).

## Supplementary Material

Crystal structure: contains datablock(s) global, I. DOI: 10.1107/S2056989015021003/hb7535sup1.cif

Structure factors: contains datablock(s) I. DOI: 10.1107/S2056989015021003/hb7535Isup2.hkl

Supporting information file. DOI: 10.1107/S2056989015021003/hb7535Isup3.cml

**Fig. 1.**
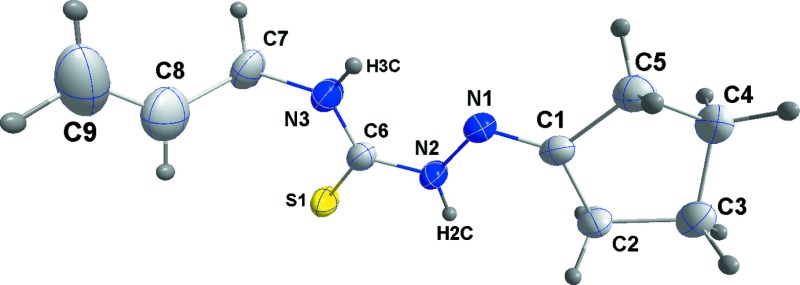
The title molecule, shown with 50% probability ellipsoids.

**Fig. 2.**
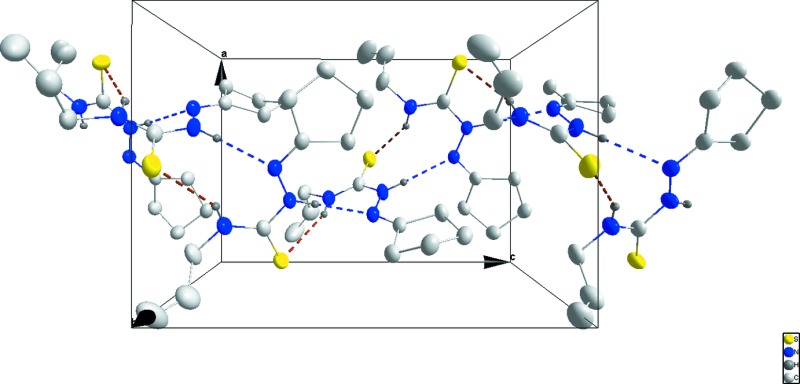
The packing in the title molecule, viewed down the b axis. N—H···N and N—H···S hydrogen bonds are shown, respectively, as blue and brown dotted lines.

. DOI: 10.1107/S2056989015021003/hb7535fig1.tif
The title mol­ecule, shown with 50% probability ellipsoids.

b . DOI: 10.1107/S2056989015021003/hb7535fig2.tif
The packing in the title mol­ecule, viewed down the *b* axis. N—H⋯N and N—H⋯S hydrogen bonds are shown, respectively, as blue and brown dotted lines.

CCDC reference: 1435175

Additional supporting information:  crystallographic information; 3D view; checkCIF report

## Figures and Tables

**Table 1:** Hydrogen-bond geometry (Å, °)

*D*—H⋯*A*	*D*—H	H⋯*A*	*D*⋯*A*	*D*—H⋯*A*
N2—H2*C*⋯N1^i^	0.91	2.29	3.194 (4)	177
N3—H3*C*⋯N1	0.91	2.25	2.642 (4)	106
N3—H3*C*⋯S1^ii^	0.91	2.49	3.310 (3)	151
C3—H3*A*⋯S1^iii^	0.99	2.86	3.663 (4)	139

Symmetry codes: (i) 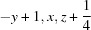; (ii) 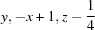; (iii) 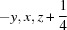.

## supplementary crystallographic information

### S1. Comment

The thiosemicarbazones comprise a class of molecules known for their diverse biological activities (Hu *et al.*, 2010; Costa *et al.*, 2015). In this context we report here the synthesis and crystal structure determination of the title compound.

In the title compound (Fig. 1), a Cremer-Pople analysis of the conformation of the 5-membered ring gave puckering parameters Q(2) = 0.380 (4) Å and φ(2) = 285.3 (6)°. The molecules pack in helical chains about the 4_1_ axis assisted by an N2—H2C···N1^i^ and an N3—H3C···S1^ii^ (i: 1 - *y*, *x*, 1/4 + *z*; ii: *y*, 1 - *x*, -1/4 + *z*) hydrogen bond between each pair of adjacent molecules (Table 1 and Fig. 2).

### S2. Experimental

The title compound was prepared according to our recently reported method (Mague *et al.*, 2014). Colourless blocks were grown from ethanol solution by slow evaporation. *M*. p. 374–375 K; 89% yield.

### S3. Refinement

H atoms attached to carbon were placed in calculated positions (C—H = 0.95 - 0.99 Å) while those attached to nitrogen were placed in locations derived from a difference map and their parameters adjusted to give N—H = 0.91 Å. All were included as riding contributions with isotropic displacement parameters 1.2 times those of the attached atoms.

### Crystal data

**Table:**

C_9_H_15_N_3_S	*D*_x_ = 1.259 Mg m^−^^3^
*M**_r_* = 197.30	Cu *K*α radiation, λ = 1.54178 Å
Tetragonal, *P*4_1_	Cell parameters from 7005 reflections
Hall symbol: P 4w	θ = 3.5–72.4°
*a* = 9.0124 (2) Å	µ = 2.42 mm^−^^1^
*c* = 12.8200 (2) Å	*T* = 150 K
*V* = 1041.28 (5) Å^3^	Block, colourless
*Z* = 4	0.21 × 0.16 × 0.10 mm
*F*(000) = 424	

### Data collection

**Table:**

Bruker D8 VENTURE PHOTON 100 CMOS diffractometer	1990 independent reflections
Radiation source: INCOATEC IµS micro–focus source	1901 reflections with *I* > 2σ(*I*)
Mirror monochromator	*R*_int_ = 0.032
Detector resolution: 10.4167 pixels mm^-1^	θ_max_ = 72.4°, θ_min_ = 4.9°
ω scans	*h* = −10→11
Absorption correction: multi-scan (*SADABS*; Bruker, 2014)	*k* = −11→11
*T*_min_ = 0.67, *T*_max_ = 0.79	*l* = −15→15
7869 measured reflections	

### Refinement

**Table:**

Refinement on *F*^2^	Hydrogen site location: mixed
Least-squares matrix: full	H-atom parameters constrained
*R*[*F*^2^ > 2σ(*F*^2^)] = 0.041	*w* = 1/[σ^2^(*F*_o_^2^) + (0.0616*P*)^2^ + 0.3211*P*] where *P* = (*F*_o_^2^ + 2*F*_c_^2^)/3
*wR*(*F*^2^) = 0.109	(Δ/σ)_max_ < 0.001
*S* = 1.10	Δρ_max_ = 0.70 e Å^−^^3^
1990 reflections	Δρ_min_ = −0.18 e Å^−^^3^
119 parameters	Absolute structure: Flack *x* determined using 826 quotients [(*I*^+^)-(*I*^-^)]/[(*I*^+^)+(*I*^-^)] (Parsons *et al.*, 2013)
1 restraint	Absolute structure parameter: 0.04 (3)

### Special details

**Table:**

Geometry. Bond distances, angles *etc*. have been calculated using the rounded fractional coordinates. All su's are estimated from the variances of the (full) variance-covariance matrix. The cell e.s.d.'s are taken into account in the estimation of distances, angles and torsion angles
Refinement. Refinement on *F*^2^ for ALL reflections except those flagged by the user for potential systematic errors. Weighted *R*-factors *wR* and all goodnesses of fit *S* are based on *F*^2^, conventional *R*-factors *R* are based on *F*, with *F* set to zero for negative *F*^2^. The observed criterion of *F*^2^ > σ(*F*^2^) is used only for calculating -*R*-factor-obs *etc*. and is not relevant to the choice of reflections for refinement. *R*-factors based on *F*^2^ are statistically about twice as large as those based on *F*, and *R*-factors based on ALL data will be even larger.

### Fractional atomic coordinates and isotropic or equivalent isotropic displacement parameters (Å^2^)

**Table:**

	*x*	*y*	*z*	*U*_iso_*/*U*_eq_	
S1	0.50830 (11)	0.10489 (10)	0.51268 (7)	0.0404 (3)	
N1	0.2886 (3)	0.4807 (3)	0.5217 (2)	0.0312 (8)	
N2	0.3701 (3)	0.3575 (3)	0.5510 (2)	0.0325 (8)	
N3	0.3362 (3)	0.2660 (3)	0.3854 (2)	0.0357 (9)	
C1	0.2584 (4)	0.5764 (4)	0.5926 (3)	0.0293 (9)	
C2	0.3050 (4)	0.5761 (4)	0.7060 (3)	0.0339 (10)	
C3	0.2402 (4)	0.7223 (4)	0.7498 (3)	0.0362 (10)	
C4	0.2242 (4)	0.8220 (4)	0.6547 (3)	0.0389 (11)	
C5	0.1737 (4)	0.7160 (4)	0.5686 (3)	0.0359 (11)	
C6	0.3975 (4)	0.2491 (4)	0.4790 (3)	0.0331 (10)	
C7	0.3533 (5)	0.1587 (5)	0.3012 (3)	0.0430 (11)	
C8	0.2503 (6)	0.0262 (6)	0.3117 (4)	0.0617 (17)	
C9	0.1462 (7)	−0.0066 (7)	0.2532 (5)	0.079 (2)	
H2A	0.41450	0.57460	0.71240	0.0410*	
H2B	0.26350	0.48910	0.74300	0.0410*	
H2C	0.41060	0.34020	0.61480	0.0390*	
H3A	0.14260	0.70480	0.78310	0.0430*	
H3B	0.30820	0.76700	0.80170	0.0430*	
H3C	0.27400	0.34430	0.37540	0.0430*	
H4A	0.32000	0.86910	0.63660	0.0470*	
H4B	0.14930	0.90050	0.66700	0.0470*	
H5A	0.06530	0.69880	0.57190	0.0430*	
H5B	0.19970	0.75490	0.49880	0.0430*	
H7A	0.45720	0.12310	0.30000	0.0520*	
H7B	0.33340	0.20860	0.23380	0.0520*	
H8	0.26760	−0.03810	0.36910	0.0740*	
H9A	0.12420	0.05410	0.19460	0.0950*	
H9B	0.08830	−0.09250	0.26690	0.0950*	

### Atomic displacement parameters (Å^2^)

**Table:**

	*U*^11^	*U*^22^	*U*^33^	*U*^12^	*U*^13^	*U*^23^
S1	0.0529 (6)	0.0352 (4)	0.0332 (4)	0.0076 (4)	−0.0021 (4)	−0.0046 (4)
N1	0.0366 (14)	0.0334 (13)	0.0235 (12)	−0.0002 (11)	−0.0003 (12)	0.0003 (12)
N2	0.0433 (16)	0.0325 (15)	0.0217 (12)	0.0033 (12)	−0.0040 (12)	−0.0021 (11)
N3	0.0413 (17)	0.0387 (17)	0.0272 (15)	0.0005 (12)	−0.0016 (13)	−0.0047 (12)
C1	0.0316 (16)	0.0328 (17)	0.0236 (16)	−0.0033 (13)	0.0013 (13)	0.0030 (13)
C2	0.0415 (19)	0.0357 (18)	0.0245 (17)	0.0010 (15)	−0.0038 (14)	0.0002 (13)
C3	0.0379 (18)	0.0401 (18)	0.0307 (17)	−0.0040 (14)	0.0039 (15)	−0.0051 (15)
C4	0.047 (2)	0.0338 (18)	0.036 (2)	0.0042 (15)	0.0050 (17)	−0.0026 (15)
C5	0.0389 (18)	0.0392 (19)	0.0297 (18)	0.0042 (15)	−0.0013 (14)	0.0033 (15)
C6	0.0372 (17)	0.0361 (18)	0.0260 (16)	−0.0053 (13)	0.0023 (14)	−0.0018 (13)
C7	0.044 (2)	0.055 (2)	0.0299 (17)	−0.0055 (17)	0.0020 (15)	−0.0131 (17)
C8	0.065 (3)	0.061 (3)	0.059 (3)	−0.002 (2)	0.005 (2)	−0.018 (2)
C9	0.075 (3)	0.090 (4)	0.073 (4)	−0.040 (3)	0.019 (3)	−0.028 (3)

### Geometric parameters (Å, º)

**Table:**

S1—C6	1.695 (4)	C8—C9	1.237 (8)
N1—N2	1.383 (4)	C2—H2A	0.9900
N1—C1	1.282 (5)	C2—H2B	0.9900
N2—C6	1.367 (5)	C3—H3A	0.9900
N3—C6	1.330 (5)	C3—H3B	0.9900
N3—C7	1.457 (5)	C4—H4A	0.9900
C1—C2	1.513 (5)	C4—H4B	0.9900
C1—C5	1.503 (5)	C5—H5A	0.9900
C2—C3	1.547 (5)	C5—H5B	0.9900
N2—H2C	0.9100	C7—H7A	0.9900
C3—C4	1.521 (5)	C7—H7B	0.9900
N3—H3C	0.9100	C8—H8	0.9500
C4—C5	1.529 (5)	C9—H9A	0.9500
C7—C8	1.519 (7)	C9—H9B	0.9500
			
N2—N1—C1	117.4 (3)	C2—C3—H3B	111.00
N1—N2—C6	119.1 (3)	C4—C3—H3A	111.00
C6—N3—C7	123.3 (3)	C4—C3—H3B	111.00
N1—C1—C2	128.4 (3)	H3A—C3—H3B	109.00
N1—C1—C5	121.7 (3)	C3—C4—H4A	111.00
C2—C1—C5	109.8 (3)	C3—C4—H4B	111.00
C1—C2—C3	104.0 (3)	C5—C4—H4A	111.00
N1—N2—H2C	127.00	C5—C4—H4B	111.00
C6—N2—H2C	114.00	H4A—C4—H4B	109.00
C2—C3—C4	104.4 (3)	C1—C5—H5A	111.00
C6—N3—H3C	118.00	C1—C5—H5B	111.00
C7—N3—H3C	118.00	C4—C5—H5A	111.00
C3—C4—C5	103.8 (3)	C4—C5—H5B	111.00
C1—C5—C4	102.9 (3)	H5A—C5—H5B	109.00
S1—C6—N2	118.9 (3)	N3—C7—H7A	109.00
N2—C6—N3	116.9 (3)	N3—C7—H7B	109.00
S1—C6—N3	124.2 (3)	C8—C7—H7A	109.00
N3—C7—C8	113.1 (3)	C8—C7—H7B	109.00
C7—C8—C9	126.7 (5)	H7A—C7—H7B	108.00
C1—C2—H2A	111.00	C7—C8—H8	117.00
C1—C2—H2B	111.00	C9—C8—H8	117.00
C3—C2—H2A	111.00	C8—C9—H9A	120.00
C3—C2—H2B	111.00	C8—C9—H9B	120.00
H2A—C2—H2B	109.00	H9A—C9—H9B	120.00
C2—C3—H3A	111.00		
			
C1—N1—N2—C6	176.4 (3)	N1—C1—C2—C3	177.8 (4)
N2—N1—C1—C2	2.3 (5)	C5—C1—C2—C3	1.3 (4)
N2—N1—C1—C5	178.5 (3)	N1—C1—C5—C4	−154.9 (3)
N1—N2—C6—S1	175.4 (2)	C2—C1—C5—C4	21.9 (4)
N1—N2—C6—N3	−3.5 (5)	C1—C2—C3—C4	−24.2 (4)
C7—N3—C6—S1	2.2 (5)	C2—C3—C4—C5	38.1 (3)
C7—N3—C6—N2	−179.0 (3)	C3—C4—C5—C1	−36.6 (3)
C6—N3—C7—C8	80.2 (5)	N3—C7—C8—C9	113.4 (6)

### Hydrogen-bond geometry (Å, º)

**Table:**

*D*—H···*A*	*D*—H	H···*A*	*D*···*A*	*D*—H···*A*
N2—H2*C*···N1^i^	0.91	2.29	3.194 (4)	177
N3—H3*C*···N1	0.91	2.25	2.642 (4)	106
N3—H3*C*···S1^ii^	0.91	2.49	3.310 (3)	151
C3—H3*A*···S1^iii^	0.99	2.86	3.663 (4)	139

Symmetry codes: (i) −*y*+1, *x*, *z*+1/4; (ii) *y*, −*x*+1, *z*−1/4; (iii) −*y*, *x*, *z*+1/4.
