# Supplementary figures and images for: Crystal structure of 1-(cyclo­pentyl­idene­amino)-3-(prop-2-en-1-yl)thio­urea
Source: Acta Crystallogr E Crystallogr Commun. 2015 Nov 11;71(Pt 12):o924–5. doi: 10.1107/S2056989015021003 (PMC4719883; doi:10.1107/S2056989015021003)

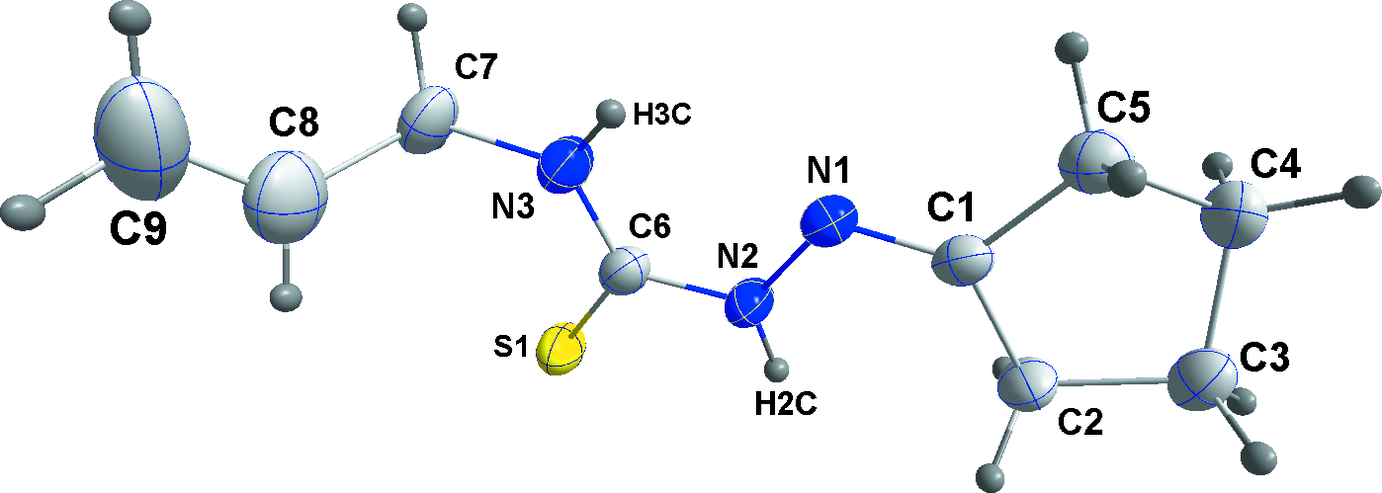

Supplement: Supplementary file 4 [file e-71-0o924-fig1.tif]

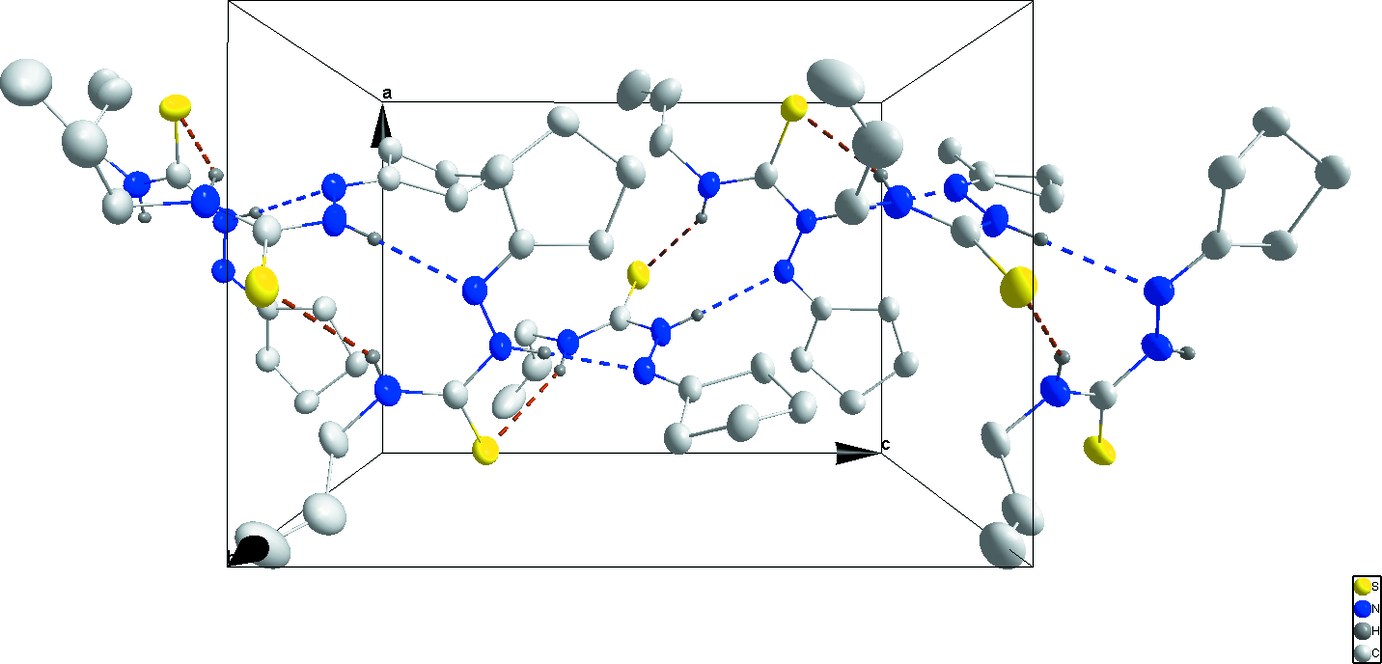

Supplement: Supplementary file 5 [file e-71-0o924-fig2.tif]
